# Occupational Screening for Tuberculosis and the Use of a Borderline Zone for Interpretation of the IGRA in German Healthcare Workers

**DOI:** 10.1371/journal.pone.0115322

**Published:** 2014-12-26

**Authors:** Anja Schablon, Albert Nienhaus, Felix C. Ringshausen, Alexandra M. Preisser, Claudia Peters

**Affiliations:** 1 University Medical Center Hamburg-Eppendorf (UKE), Center of Excellence for Health Services Research in Nursing (CVcare), Hamburg, Germany; 2 Institute for Statutory Accident Insurance and Prevention in the Health and Welfare Services (BGW), Principles of Prevention and Rehabilitation Department (GPR), Hamburg, Germany; 3 Hanover Medical School, Department of Respiratory Medicine, Hanover, Germany; 4 University Medical Center Hamburg-Eppendorf (UKE), Institute for Occupational and Maritime Medicine, Hamburg, Germany; University Hospital Schleswig Holstein, Germany

## Abstract

**Introduction:**

Healthcare workers (HCWs) in low incidence countries with contact to patients with tuberculosis (TB) are considered a high-risk group for latent TB infection (LTBI) and therefore are routinely screened for LTBI. The German Occupational TB Network data is analyzed in order to estimate the prevalence and incidence of LTBI and to evaluate putative risk factors for a positive IGRA and the performance of IGRA in serial testing.

**Methods:**

3,823 HCWs were screened with the Quantiferon Gold in Tube (QFT) at least once; a second QFT was performed on 817 HCWs either in the course of contact tracing or serial examination. Risk factors for a positive QFT were assessed by a questionnaire.

**Results:**

We observed a prevalence of LTBI of 8.3%. Putative risk factors for a positive QFT result were age >55 years (OR 6.89), foreign country of birth (OR 2.39), personal history of TB (OR 6.23) and workplace, e.g. internal medicine (OR 1.40), infection ward (OR 1.8) or geriatric care (OR 1.8). Of those repeatedly tested, 88.2% (721/817) tested consistently QFT-negative and 47 were consistently QFT-positive (5.8%). A conversion was observed in 2.8% (n = 21 of 742 with a negative first QFT) and a reversion occurred in 37.3% (n = 28 of 75 with a positive first QFT). Defining a conversion as an increase of the specific interferon concentration from <0.2 to >0.7 IU/ml, the conversion rate decreased to 1.2% (n = 8). Analogous to this, the reversion rate decreased to 18.8% (n = 9).

**Discussion:**

In countries with a low incidence of TB and high hygiene standards, the LTBI infection risk for HCWs seems low. Introducing a borderline zone from 0.2 to ≤0.7 IU/ml may help to avoid unnecessary X-rays and preventive chemotherapy. No case of active TB was detected. Therefore, it might be reasonable to further restrict TB screening to HCWs who had unprotected contact with infectious patients or materials.

## Introduction

The risk of latent tuberculosis infection (LTBI) and active tuberculosis (TB) for healthcare workers (HCWs) is well established. In line with the decrease of TB incidence in countries like Germany, the risk of TB infection for HCWs is likely to decrease as well. However, an extra risk due to working in healthcare seems to remain even in high-income countries with sophisticated hygiene standards [1]–[6]. Therefore, TB screening for HCWs is performed in order to prevent nosocomial transmission from HCWs to patients and in order to detect and treat recent LTBI in HCWs [7]. Screening can be performed either as pre-employment screening, as repeated routine screening of high-risk groups or as contact tracing after accidental exposure to TB patients or infectious materials. Pre-employment screening is performed in order to prevent the importation of TB into the healthcare system. This is particularly important when the new recruits belong to high-risk groups for TB such as migrants from high incidence countries or persons with a personal history of TB. As TB incidence in Germany is low (5.3/100,000) [8], there is no general regulation on pre-employment screening. In accordance with German Occupational Safety and Health (OSH) regulations [9], TB screening is performed routinely as repeated screening of high-risk groups, e.g. HCWs who have regular contact with contagious TB patients or material. All other HCWs are only screened after accidental contact.

These HCW screenings were performed with the tuberculin skin test (TST) for many years. The TST has several weaknesses, the most important ones being cross-reactivity with BCG vaccination, time investment and non-compliance in TST programs, and booster phenomena in serial testing due to its intradermal application. Interferon-gamma release assays (IGRA) are a promising tool to overcome these problems [10]. For several years, two Interferon-gamma release assays (IGRA) have been commercially available: the ELISA-based Quantiferon Gold in Tube (QFT) and the ELISPOT-based T.SPOT.TB. Data on their performance in TB screening of HCWs has become available from different countries, including Germany, Portugal, France, the USA and Canada. They are currently being evaluated for use in serial TB screenings of HCWs [10]–[13]. So far, the variability of the IGRA in serial testing is not well understood. Several studies have reported high rates of IGRA conversions and reversions [11]–[16]. Taking into account these high conversion and reversion rates in low TB incidence countries, the interpretation of test results in serial testing has become an important issue.

When IGRA became commercially available, the German Occupational TB Network of occupational physicians was set up in order to systematically collect the results of TB screening of HCWs with IGRA in the scope of German OSH legislation. In particular, the prevalence and incidence of LTBI in HCWs and risk factors for a positive IGRA are assessed. Within this data presentation, special emphasis is placed on the performance of IGRA in the serial testing of HCWs. The effect of introducing a borderline zone on the variability of IGRA results is analyzed in this context.

## Materials and Methods

### 2.1 Study design and subjects

The convenience sample of this cohort study consists of HCWs from hospitals, nursing homes and out-patient care units, which participated in TB screening in the context of the German Occupational TB Network from January 2006 to December 2013. The participating occupational physicians selected the HCWs to be screened following German occupational and health regulation [9]. All HCWs with regular contact to TB patients, regardless of protected or unprotected contact, are screened at intervals from annually to every third year, depending on the risk assessment of the physician. The participation rate in the screenings for those who fulfill the inclusion criteria is close to 100% as, until 2014, an OSH regulation did not allow HCWs to perform tasks involving infection risks unless the occupational physician certified the HCW's fitness for such tasks. In addition, voluntary screenings are offered to HCWs after accidental exposure on wards where normally no TB patients are treated. All participants with a positive IGRA at baseline or showing a conversion were offered a clinical and radiological examination to rule out active TB. LTBI is defined as a positive IGRA in the absence of medical symptoms and signs of an active TB in the chest X-ray.

The repeated screening included a second QFT and a second standardized questionnaire. The same selection criteria applied as for the first IGRA. The reason for retesting (after accidental contact to TB-cases or during routine screening of high risk groups) was defined by the occupational physicians following the occupational and health regulation and not by a strict study protocol.

### 2.2. Questionnaire items

Information on the following variables was collected by the occupational physicians using a standardized questionnaire: age, gender, reason for testing, occupational exposure to TB, time spent working in healthcare sector, personal and family history of TB, country of birth, previous TST results, job title and workplace. At the time of the second IGRA, HCWs were asked whether preventive chemotherapy was offered and taken after the baseline IGRA. Furthermore, chest radiograph findings and BCG vaccination by clinical inspection and vaccination records were determined by the occupational physicians.

### 2.3. Diagnostic methods

The QFT was administered as specified by the manufacturer. The test was considered positive if INF-γ was ≥0.35 IU/ml after correction for the negative control. Concentrations of above 10 IU/ml were set at 10 IU/ml because of imprecision of measurement at these high concentrations [17]. For the serial testing data analysis, a borderline zone from 0.2 to <0.7 IU/ml was assumed, as proposed by several studies of serial QFT testing in HCWs [6], [11], [14], [18]. The upper limit of 0.7 IU/ml was assumed because this is twice the cut-off of 0.35 IU/ml. In reference to the definition of the borderline zone, a QFT result of <0.2 was considered negative, a result of 0.2 to ≤0.7 IU/ml was considered a borderline result and a QFT result of >0.7 IU/ml was considered positive. Alternatively, a borderline zone from 0.1 to ≤1.0 IU/ml was tested. A conversion is defined as a change of the QFT result from negative to positive or as an increase of the specific Interferon gamma concentration from <0.2 (0.1) to>0.7 (1.0) IU/ml. Accordingly, a reversion is defined as a change of the QFT result from positive to negative or as a decrease of the Interferon-gamma concentration from >0.7 (1.0) to <0.2 (0.1) IU/ml.

### 2.4. Statistical analysis

Data analysis was performed using SPSS Version 21. Chi-square tests were used for categorical data. Adjusted odds ratios (OR) and 95% confidence intervals (CI) were calculated for risk factors for a positive QFT using the conditional logistic regression model. Model building was performed backwards, using the change criteria for variable selection. Baseline INF-γ concentration was categorized in small increments in order to observe the increment at which the highest change in conversion and reversion rates occurs.

### 2.5. Ethical statement

The study protocol was approved by the ethics committee of the Hamburg Medical Council. The study was performed in accordance with the ethical standards laid down in the 1964 Declaration of Helsinki and its later amendments. All participants gave their written informed consent prior to their inclusion in the study.

## Results

### 4.1. Prevalence and risk factors for positive IGRA results

**Figure 1:**
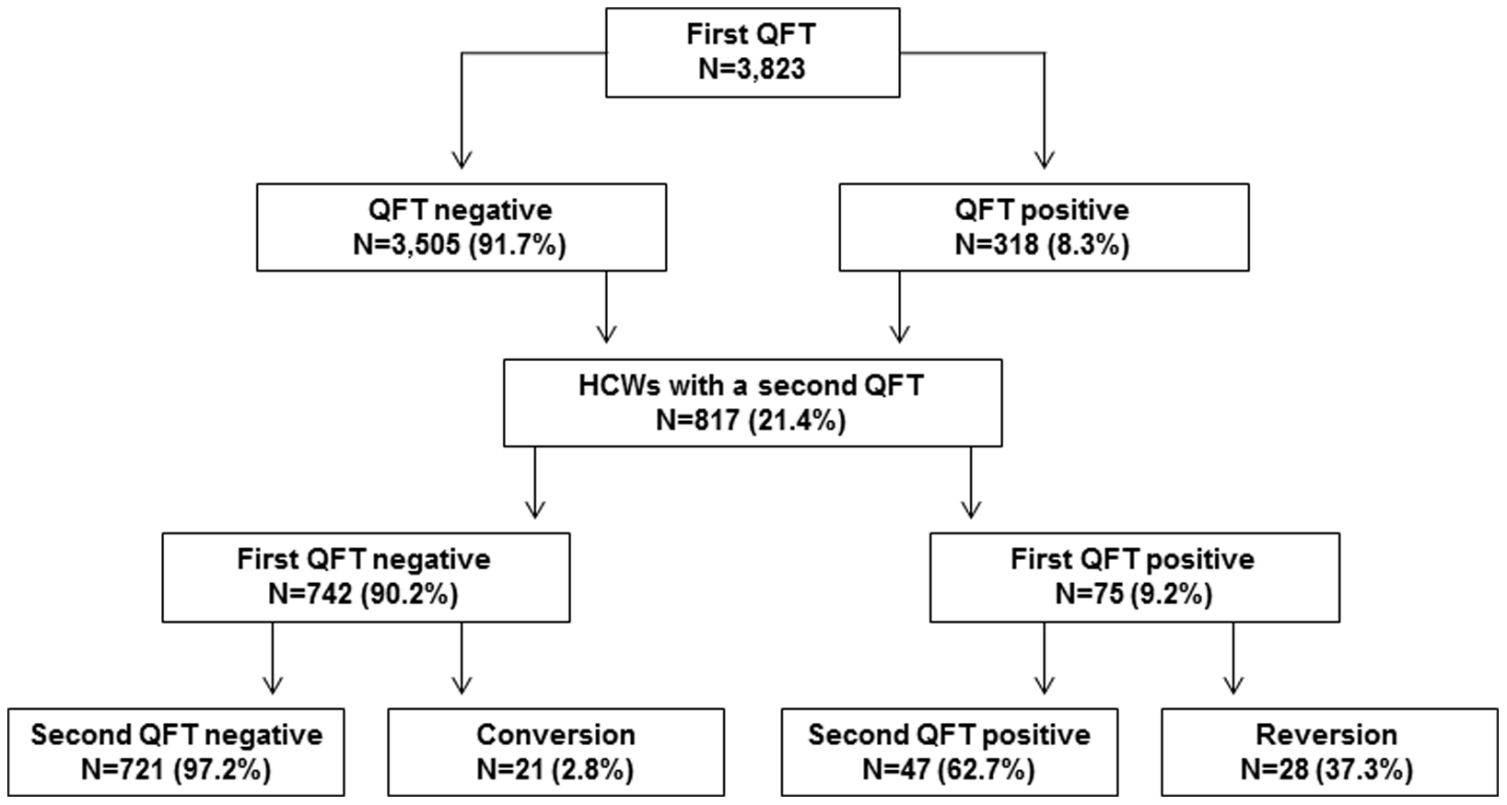
Flowchart: Study population and QFT results.

The study population comprises 3,823 HCWs from 32 hospital, nursing homes and out-patient care units. A total of 318 positive QFT results (8.3%) were observed at baseline (Fig. 1). The majority of the participants (77.4%) were female and the mean age was 38.9 years (SD 12.5). 92% of the 589 foreign born participants were born in countries with high incidence of TB. More than half of the study population worked as a nurse (51.3%) with a prevalence of 8.1% QFT-positive (Table 1).

**Table 1:** Description of the study population and frequencies and adjusted odds ratios (OR) including 95% confidence intervals (95% CI) for covariates associated with positive QFT results.

Covariates		QFT -	QFT +	OR *	95% CI
**Age**	**N (Col-%)**	**N (Row-%)**	**N (Row-%)**		
<25 years	510 (13.3)	496 (97.3)	14 (2.7)	1	-
25–35 years	926 (24.2)	878 (94.8)	48 (5.2)	**1.72**	**1.71–1.72**
35–45 years	1039 (27.2)	965 (92.9)	74 (7.1)	**2.11**	**2.11–2.12**
45–55 years	975 (25.5)	868 (89.0)	107 (11.0)	**3.52**	**3.51–3.54**
>55 years	373 (9.8)	298 (79.9)	75 (20.1)	**6.89**	**6.87–6.91**
**Gender**					
Female	2959 (77.4)	2716 (91.8)	243 (8.2)	1	
Male	864 (22.6)	789 (91.3)	75 (8.7)	1.29	1.293–1.298
**Country of birth**					
Germany	3234 (84.6)	3012 (93.1)	222 (6.9)	1	
Foreign-born	589 (15.4)	493 (83.7)	96 (16.3)	**2.39**	**2.38–2.39**
**TB in own history**					
No	3788 (99.1)	3485 (92.0)	303 (8.0)	1	
Yes	35 (0.9)	20 (57.1)	15 (42.8)	**6.23**	**6.23–6.28**
**TST in history**					
no TST	1348 (35.5)	1254 (93.0)	94 (7.0)	1	
Negative	1635 (42.8)	1544 (94.4)	91 (5.6)	**0.74**	**0.737–0.74**
Positive	840 (22.0)	707 (84.2)	133 (15.8)	**1.99**	**1.99–2.0**
**Workplace**					
Other clinical wards	610 (16.0)	577 (94.6)	33 (5.4)	1	
Internal medicine	1286 (33.6)	1190 (92.5)	96 (7.5)	**1.40**	**1.40–1.41**
Admission ward	244 (6.4)	231 (94.7)	13 (5.3)	**0.90**	**0.89–0.91**
Infection ward	389 (10.2)	355 (91.3)	34 (8.7)	**1.76**	**1.75–1.76**
Geriatric care	449 (11.7)	404 (90.0)	45 (10.0)	**1.98**	**1.98–1.99**
Rad/Lab/Path	293 (7.7)	252 (86.0)	41(14.0)	**2.35**	**2.34–2.35**
Administration	117 (3.1)	101 (86.3)	16 (13.7)	**2.89**	**2.88–2.91**
ICU	435 (11.4)	395 (90.8)	40 (9.2)	**1.50**	**1.50–1.51**
**Profession**			Not included in the final model		
Physicians	583 (15.2)	538 (92.3)	45 (7.7)	0.82	0.82–0.824
Nurses	1962 (51.3)	1804 (91.9)	158 (8.1)	0,94	0.938–0.944
Administration staff	267 (7.0)	229 (85.8)	38 (14.2)	1.16	1.157–1.17
Technicians and special ward staff	222 (5.8)	200 (90.1)	22 (9.9)	0.57	0.56–0.57
Other	302 (7.9)	277 (91.7)	25 (8.3)	1	
Trainees	177 (4.6)	174 (98.3)	3 (1.7)	0.40	0.36–0.37
Therapist/Auxiliaries	310 (8.1)	283 (91.3)	27 (8.7)	0.92	0.91–0.92
**Reason for testing**			Not included in the final model		
Serial examination	2533 (66.3)	2310 (91.2)	223 (8.8)	1	
Contact tracing	1290 (33.7)	1195 (92.6)	95 (7.4)	0.90	0.90–0.97
**BCG vaccination**			Not included in the final model		
No	2084 (54.4)	1909 (91.6)	175 (8.4)	1	
Yes	1739 (45.5)	1596 (91.8)	143 (8.2)	0.88	0.882–0.89

*The final multivariate logistics model includes the variables age, gender, country of birth, TB in own history, workplace, TST.

Rad/Lab/Path  =  Radiology, Laboratory, Pathology.

Risk factors for a positive QFT result were an age of >55 years (OR 6.89, 95% CI 6.87–9.91), being foreign born (OR 2.39, 95% CI 2.38–2.39), TB in the individual's own history (OR 6.23, 95% CI 6.23–6.28) and workplace (Table 1). No statistically significant association was observed for the criterion of profession and reason for testing. No case of active TB was detected during the baseline screening.

### 4.2. IGRA variability in serial testing data

A second QFT was performed on 817 HCWs. The average time span between the two QFT tests was 13.1 months (minimum 7 days, maximum 48.6 month) and 12.8 months (minimum 7 days, maximum 33.5 months) for the follow-up period of the 75 HCWs with a positive QFT at baseline. The duration between the two tests did not differ depending on reversions, conversions or stable results. Duration between tests: both tests negative 13.1 months (minimum 1 week, maximum 48.5 months); both tests positive 12.8 months (minimum 1.9 months, maximum 33.5 month); reversion 12.6 months (minimum 2.8 months, maximum 32.2 months); conversion 12.6 months (minimum 7.5, maximum 46.7). The nonparametric test for the comparison of the distribution of the duration in these four groups was not statistically significant (Kruskal Wallis p = 0.27). Chemoprevention was recommended for 14 participants with a positive QFT at baseline but only one participant accepted and completed chemoprevention. The proportion of positive QFT in this subgroup was similar to that in the total group (9.2 versus 8.3) (Fig. 1). Of those repeatedly tested, 721 out of 742 individuals were consistently QFT-negative (97.2%) and 47 out of 75 (62.7%) were consistently QFT-positive (Table 2). The probability of two positive QFT tests increased with age from 2% for those below 25 years to 18.5% in HCWs with an age of 55+ years. No association with age is apparent for the conversion und reversion rates. The reversion rate for foreign-born HCWs was higher than for German-born HCWs (7.8 versus 2.7%). The highest conversion rate was observed in HCWs from infections wards (13.6%). However, this is based on few observations (n = 3).

**Table 2:** Serial testing results of the study population (n = 817) with two IGRA results.

Variables	N	Col-%	Conversion	Reversion	Both +	Both -
**Age** *			n (%)	n (%)	n (%)	n (%)
<25 years	101	12.4	2 (2.0)	2 (2.0)	2 (2.0)	95 (94.1)
25–35 years	176	21.5	7 (4.0)	8 (4.5)	5 (2.8)	156 (88.6)
36–45 years	217	26.6	6 (2.8)	7 (3.2)	6 (2.8)	198 (91.2)
46–55 years	242	29.6	6 (2.5)	6 (2.5)	19 (7.9)	211 (87.2)
≥55 years	81	9.9	-	5 (6.2)	15 (18.5)	61 (75.3)
**Gender** *						
Female	665	81.4	17 (2.6)	25 (3.8)	35 (5.3)	588 (88.4)
Male	152	18.6	4 (2.6)	3 (2.0)	12 (7.9)	133 (87.5)
**Country of birth** *						
Germany	701	85.5	17 (2.4)	19 (2.7)	37 (5.3)	628 (89.6)
Foreign born	116	14.2	4 (3.4)	9 (7.8)	10 (8.6)	93 (80.2)
TB history*						
No	811	99.3	21 (2.6)	28 (3.5)	43(5.3)	719 (99.7)
Yes	6	0.7	-	-	4 (66.7)	2 (33.3)
**Known contact with index case between tests** *						
No	625	77.9	15 (2.4)	24 (3.8)	38 (6.1)	548 (87.7)
Yes	177	22.1	6 (3.4)	4 (2.3)	7 (4.0)	160 (90.4)
**Profession** *						
Administrator	185	22.6	3 (1.6)	5 (2.7)	11 (5.9)	166 (89.7)
Auxiliary, cleaning staff	31	3.8	1 (3.2)	1 (3.2)	-	29 (93.5)
Technician laboratory, etc.)	45	5.5	1 (2.2)	3 (6.7)	3 (6.7)	38 (84.4)
Nurse	449	55.0	12 (2.7)	17 (3.8)	25 (5.6)	395 (88.0)
Doctor	107	13.1	4 (3.7)	2 (1.9)	8 (7.5)	93 (86.9)
**Workplace** *						
Admission ward	64	7.8	2 (3.1)	3 (4.7)	3 (4.7)	56 (87.5)
Infection ward	22	2.7	3 (13.6)	-	1 (4.5)	18 (81.8)
Pulmonology ward	77	9.4	2 (2.6)	1 (1.3)	4 (5.2)	70 (90.9)
Geriatric care	88	10.8	1 (1.1)	3 (3.4)	5 (5.7)	79 (89.8)
Laboratory	38	4.7	1 (2.6)	4 (10.5)	4 (10.5)	29 (76.3)
Radiology/Pathology	37	4.5	3 (8.1)	-	5 (13.5)	29 (78.4)
Internal medicine	164	20.1	7 (4.3)	4 (2.4)	12 (7.3)	141 (86.0)
Surgical ward	76	9.3	-	3 (3.9)	2 (2.6)	71 (93.4)
Technicians	77	9.4	1 (1.3)	4 (5.2)	5 (6.5)	67 (87.0)
Other	174	21.3	1 (0.6)	6 (3.4)	6(3.4)	161 (92.5)

*Colum row

The probability of conversion and reversion depended on the Interferon-gamma concentration in the baseline QFT (Table 3). If a simple dichotomous approach (negative to positive and vice versa) was chosen, a conversion was observed in 2.8% (n = 21 out of 742) and a reversion in 37.3% of the HCWs (28 out of 75) with a positive QFT at baseline. Conversion occurred in 2.6% of the 612 HCWs with an Interferon-gamma concentration at baseline below <0.1 IU/ml but increased to 6.0% of the 50 HCWs with an INF-concentration near the cut-off (0.2 to <0.35 IU/ml). A reversion occurred in 64.7% of the 17 HCWs with an IFN-concentration near the cut-off (0.35 to <0.5 IU/ml). The reversion rate dropped to 37.5% with a baseline concentration between >1 to 3 IU/ml and to 5.2% with a baseline concentration >3 IU/ml (Table 3).

**Table 3:** Results of 2nd QFT depending on INF-γ concentration in first QFT.

1st QFT	2nd QFT					
	Negative		Positive		Total	
	N	%	N	%	N	%
<0.1 IU/ml	596	97.4	16	2.6	612	74.9
0.1-<0.2 IU/ml	78	97.5	2	2.5	80	9.8
0.2-<0.35 IU/ml	47	94.0	3	6,0	50	6,1
**Neg. 1st QFT**	**721**	**97.1**	**21**	**2.9**	**742**	**90.8**
0.35-<0.5 IU/ml	11	64.7	6	35.3	17	2.1
0.5-<0.7 IU/ml	5	50.0	5	50	10	1.2
0.7-1.0 IU/ml	2	40.0	3	60.0	5	0.6
>1–3 IU/ml	9	37.5	15	62.5	24	2.9
>3 IU/ml	1	5.2	18	94.7	19	2.3
**Pos. 1st QFT**	**28**	**37.3**	**47**	**62.7**	**75**	**9.2**
**All**	749	91.7	68	8.3	817	100.0

**Figure 2:**
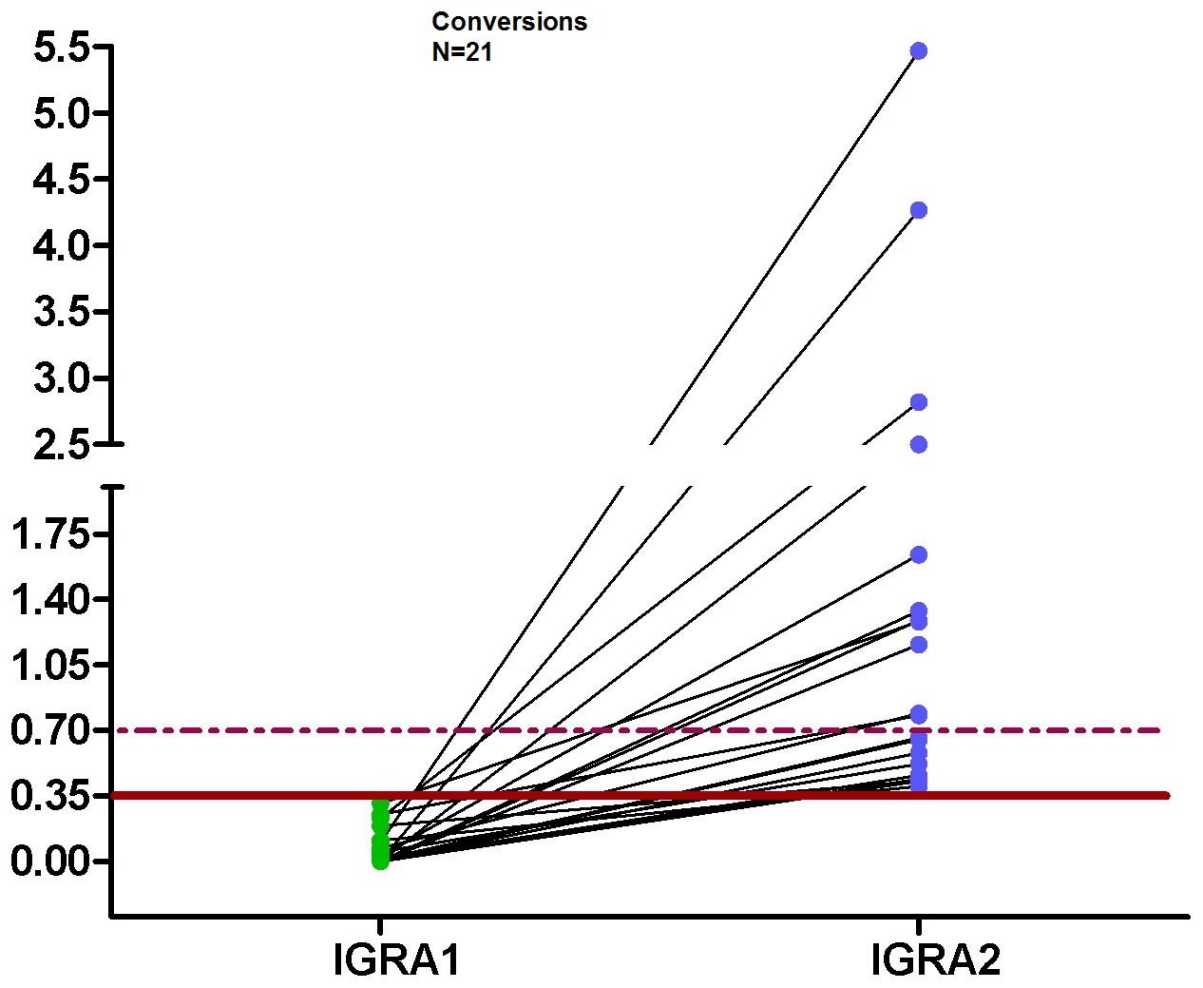
Dot plots of individual responses to QFT for conversion after the second test. The continuous line represents the cut-off 0.35 IU/ml and the dashed lines represent the borderline zone from 0.2 to <0.7 IU/ml for IFN-γ.

**Figure 3:**
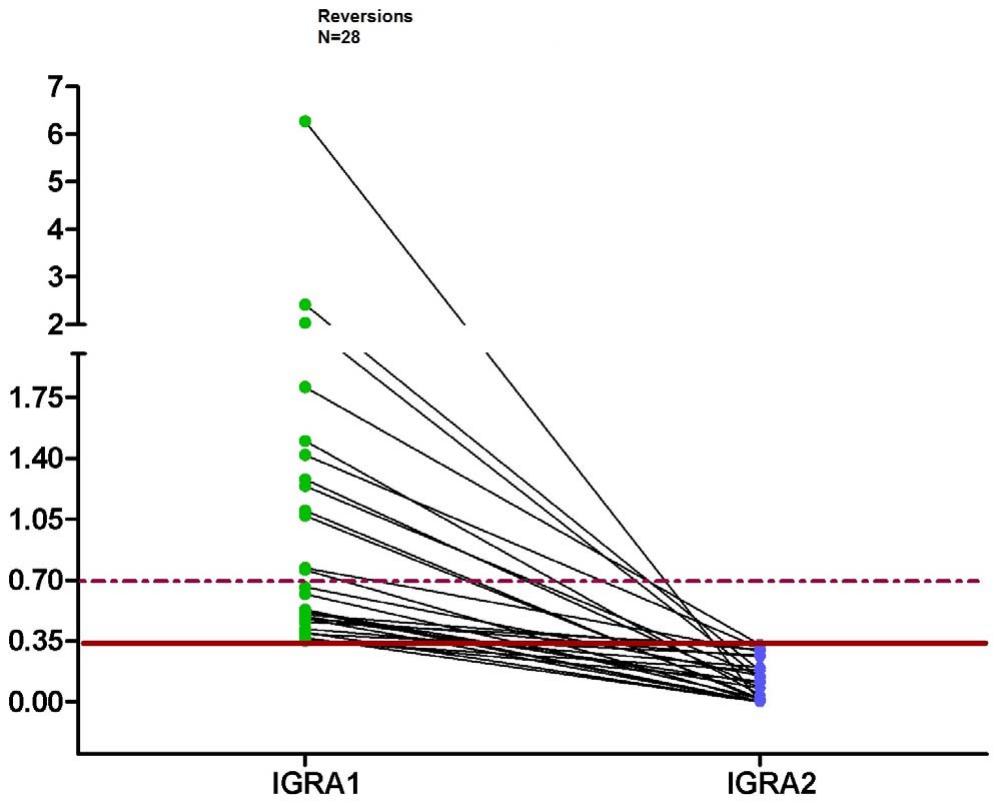
Dot plots of individual responses to QFT for reversion after the second test. The continuous line represents the cut-off 0.35 IU/ml and the dashed lines represent the borderline zone from 0.2 to <0.7 IU/ml for IFN-γ.

If the definition of conversion and reversion was limited to those with a baseline and follow-up concentration of the QFT outside of the borderline zone, i.e. <0.2 or >0.7 IU/ml, the conversion rate decreased from 2.8% (n = 21) to 1.2% (n = 8) (Figs. 1 and 2) and the reversion rate decreased to 18.8% (n = 9) (Fig. 3). Changing the upper limit of the borderline zone to 1.0 or 3.0 IU/ml further decreased the conversion rate to 1.0 or 0.4% (n = 7 or 3) and the reversion rate to 18.6 or 11.1% (n = 8 or 3) (no table). Assuming that a positive QFT within the borderline zone does not indicate LTBI or active TB, X-ray and chemoprevention could be spared depending on the upper limit of the borderline zone (0.7, 1.0, 3.0 IU/ml) in 13 (61.9%), 14 (66.7%) or 18 (85.7%) HCWs (out of 21) who otherwise need X-ray and chemoprevention. Again, no active TB was detected during the follow-up screening.

## Discussion

### 5.1. Prevalence and risk factors for a positive IGRA

We present the data from our cohort on routine QFT testing of German HCWs in the context of the German Occupational TB Network. We found an overall prevalence of positive QFT of 8.3%, which is much lower than the rate of positive TST (24–50%) in HCWs [19]-[21]. A positive QFT depended on age, personal history of TB, origin and workplace. Regarding the group of unexposed trainees, the prevalence of LTBI was low (1.7%). This low prevalence is confirmed by three other studies that found prevalence rates of 2.1% in German trainees and 0.4% in Italian healthcare students [22], [23]. A second study in Italian healthcare students found a prevalence of positive TST of 3.8% [24]. We found a lower prevalence rate than a recent French study (22.2%) [25] and a Portuguese study (29.5%) [26], but higher rates than two studies from Norway [27] and Denmark (3.4% and 1%) [28], respectively. Moreover, Fong et al. observed a prevalence of a positive QFT of 6.6% in the US [29]. In our analysis, the prevalence of a positive QFT was associated with working in any kind of department with a likelihood of contact with TB patients, e.g. infection wards (OR 1.76) or Radiology/Laboratory/Pathology (OR 2.35) but also in wards with unknown TB contacts like internal wards (OR 1.4) or geriatric care (OR 1.98). In contrast to our findings, Rafiza and Rampal observed an increased risk of LTBI for workers in emergency wards [30] and Franchi et al. found that TST conversion was associated with working in an obstetric emergency room and ambulatory discharge [31]. Several European HCW studies found no association between positive QFT and workplace [21], [25], [32], [33]. The high rate of positive IGRA (14.2%) in administrative staff seems astonishing, but the occupational physician who was responsible for the decision about the screening saw these employees as being at a certain risk. Otherwise these workers would not have been eligible for the screening. An explanation for this high prevalence may be that 65/117 (55.6%) of the administrative staff work in places with routine screening for TB performed regularly and 18/117 (15.4%) were tested after contact with TB. It could not be verified, however, whether all these employees were actually exposed to TB patients or infectious material. None of the retested participants belonged to the group of administrative staff.

The effect of introducing IGRA in TB screening in low-incidence countries is likely to reduce the number of X-rays that would be needed for the exclusion of active TB or preventive chemotherapy if the decision is based on the TST. As no TST was performed within our study, no head-to head comparison with QFT results was possible. However, in those with a prior positive TST, the confirmation rate was 15.8% (133/840). In a combined cohort of HCWs from Portugal, France and Germany, 40.2% HCWs had a positive TST that was not confirmed by an IGRA [20]. The proportion of HCWs with a BCG vaccination was 45.5 to 100% in these populations.

### 5.2. IGRA variability and interpretation of the results

So far, the variability of IGRA in serial testing is not well understood and it is a challenge to interpret the results when the IGRAs are used for repeated, e.g. annually routine screening of high-risk groups [34]. Four literature reviews have covered the topic of IGRA variability in serial testing of HCWs so far [10], [11], [35], [36] and concluded that reversion of positive IGRA to negative IGRA occurs more often than conversion from negative IGRA to positive IGRA. More importantly, the probability of conversion or reversion depends on the quantitative results of the first IGRA. Therefore, a borderline zone may be helpful in order to distinguish true conversion and reversion from variations caused by chance, i.e. inherent within subject and/or test variability [6]. Two new large studies from the US recently covered this topic. Dorman and colleagues determined the performance characteristics of the IGRA for serial testing in 2,563 HCWs undergoing occupational TB screening. They found higher conversion rates of 6.1% (QFT) and 8.3% (T-SPOT) compared to the TST (0.9%). In addition, 76.4% of the conversions were negative when they were retested after 6 months. Therefore the authors came to the conclusion that most conversions among HCWs in low TB-incidence countries appear to be false positive and repeated testing of apparent converters is warranted. They also raised the question about the usefulness of routine serial testing [12]. Slater et al. evaluated the short-term reproducibility of QFT in a cohort of 9,153 HCWs in the US as well. They found a high reversion rate of 64.8% and conversion rate of 4.4%. The later was higher than the expected 0.4% based on previous TST screenings in their institution [13]. Thus, they stated that conversions in low-risk population should be interpreted with caution.

We found a QFT conversion rate of 2.8% and a reversion rate of 37.3% among repeatedly tested HCWs if using the dichotomous definition of a positive test result. On application of a borderline zone from 0.2 to <0.7 IU/ml, conversions decreased to 1.1% and reversions to 18.8%, which seems to be more realistic than the results of the dichotomous approach. In this regard, Nienhaus and Torres examined this borderline zone among Portuguese healthcare workers and concluded that using a borderline from 0.2 to <0.7 IU/ml minimized the conversion and reversion rates in low-incidence countries and gave a more realistic estimation of conversions and reversions [6].

Fong et al. found that 71% of conversions in a low-risk group had Interferon-gamma concentrations ≤1.0 IU/ml and 36% were right around the cut-off of 0.35 to <0.5 IU/ml [29]. They recommended extending the range of the borderline zone to 0.1–1.0 IU/ml. Joshi and colleagues suggested extending the borderline to 2.0 IU/ml as all reversions in their study had concentrations below 2.0 IU/ml in the first IGRA [37]. Using a borderline between 0.2 and <1.0 IU/ml, which is the upper limit of the borderline zone proposed by Fong [29] or close to the 1.1 IU/ml proposed by Thanassi et al. [38], the conversion and reversion rate in our study decreased to 1.0% for conversions and 18.6% for reversions. Our data does not suggest reducing the lower limit of the borderline zone to 0.1 IU/ml as we observed the same conversion rate for those HCWs with a baseline Interferon-gamma concentration between 0.1 and <0.2 IU/ml as in those with a concentration <0.1 IU/ml. Furthermore, extending the borderline zone increases the number of HCWs with QFT results in the borderline zone and, therefore, increases the number of ambiguous test results. It should also be considered that the introduction of a borderline zone has certain disadvantages. Using a borderline zone reduces the sensitivity of the QFT for active TB and for LTBI, as was shown in a Portuguese study [26]. Overall, the positive predictive value (PPV) of the IGRA for disease progression was estimated to be 2.7%. The pooled PPV increased to 6.8% when only high-risk groups were considered [39]. The progression rate in HCWs seems to be lower. In Portugal a progression rate of 0.4% was observed in IGRA-positive HCWs [26]. In our German Occupational TB Network study, no progression from LTBI to active TB was found. As we only have a complete follow-up for participants with a second IGRA, a safe statement about TB progression risk cannot be made. A complete follow-up is only available for 75 HCWs with a positive first IGRA (see Fig. 1). The mean follow-up period for these 75 HCWs was 12.8 months (minimum 0 and maximum 33.5 months). Therefore, no conclusion about disease prediction can be drawn from our data. However, we recommend that HCWs with test results falling into the borderline zone should not be considered for preventive chemotherapy. Considering the high reversion rate even in those with a QFT above 0.7 IU/ml, it might be reasonable to perform a second IGRA in all HCWs for whom chemoprevention is considered. Chemoprevention might be considered for HCWs with suspected recent infection, but neither our study nor any other publication yields evidence in favor or against this approach. The effectiveness of chemoprevention in HCWs should therefore be studied in future.

In the pre-IGRA era, HCWs with a positive TST in their history had to be x-rayed every time they underwent a new TB screening. The high reversion rates we observed and which are described in literature indicate that a similar approach, i.e. once IGRA–positive, an X-ray is performed in all consecutive screenings, is not warranted. By calculating conversion and reversion rates depending on the concentration in the first IGRA, we tried to identify HCWs who can be spared from X-rays for the exclusion of active TB. If the conversion rate in HCWs with a QFT between 0.2 and <0.35 IU/ml is, for example, 50%, it seems evident that the likelihood of a recent infection is low. Therefore, we suggest not performing an X-ray in this constellation. The same is applicable for reversion. If the likelihood of a reversion is about 50%, as observed in those with a first QFT between 0.35 and 0.7 IU/ml, we do not think that an X-ray is necessary to exclude active TB.

Therefore, it seems safe to apply a borderline zone for the interpretation of the QFT and to forgo chest X-rays for those with a positive QFT between 0.35 and 0.7 or 1.0 IU/ml when no clinical symptoms are apparent and no particular intensive exposure is known. In addition, it seems reasonable to retest HCWs with a positive IGRA in history as the likelihood of a reversion is high and no X-ray for the exclusion of active TB is needed in this circumstance. This approach will further reduce the use of X-ray in the serial testing of HCWs. However, this approach needs to be scrutinized as no data is available concerning the progression risk after the reversion of an IGRA result. In addition, it should be mentioned that in countries with no BCG vaccination and consecutively low rates of positive TST results, the benefits of IGRA-based screening compared to TST-based screening may be limited. Adherence to TST screening in the serial testing of HCWs as it is proposed in Canada may thus be a prudent approach as long as the variability of the IGRAs is not completely understood [12], [14], [40].

It might even be discussed whether routine TB screening of HCWs in countries with low TB incidence and high hygiene standards might be abundant. The seemingly low acceptance rate of chemopreventive treatment (one out of 14) might even further support this argument. However, it should be kept in mind that about 90 cases of active TB in HCWs are observed in Germany each year [41], although we do not know how many of these TB cases are detected because of TB screening in HCWs.

### 5.3. Limitations

Our study has some limitations. We analyzed the data from a convenient sample of routine screenings of HCWs by occupational physicians in accordance with German Occupational Safety and Health (OSH) regulations. These screenings did not follow a strict study protocol according to the exact time schedule for the screening intervals and the selection of high-risk groups or close contacts for the screening. A more liberal testing approach may have been applied as a result. Therefore, a selection bias cannot be excluded and the results of our study are typical for HCWs screened on a regular basis in Germany and not those without regular contact to TB patients. This also may explain the surprising result that working in admission wards was protective in our study.

### 5.4. Conclusions

In countries with a low incidence of TB and high hygiene standards, the infection risk for HCWs seems to be low. Introducing a borderline zone for the interpretation of IGRA results there may help to avoid unnecessary X-rays and preventive chemotherapy. As no case of active TB was observed in our study, the German OSH regulation which restricts TB screening to HCWs who had known contact with infectious patients or materials seems to be corroborated. Further studies are needed to verify if the screening could even be restricted to those HCWs with unprotected accidental contact to TB patients or materials. As the reversion rate of the QFT is higher than expected, instead of performing X-ray in HCWs with a positive IGRA in history as it was performed in TST-based screenings, these HCWs should be retested with an IGRA if a new routine screening is scheduled. The limitations of our study considered a large prospective study over a long follow-up period with systematic follow-up and it is necessary to define inclusion and exclusion criteria in order to examine whether TB screening of HCWs in a country with low TB incidence and high hygiene standards is effective.
