# Correction: Occupational Screening for Tuberculosis and the Use of a Borderline Zone for Interpretation of the IGRA in German Healthcare Workers

**DOI:** 10.1371/journal.pone.0142541

**Published:** 2015-11-05

**Authors:** Anja Schablon, Albert Nienhaus, Felix C. Ringshausen, Alexandra M. Preisser, Claudia Peters, The PLOS ONE Staff

Following publication of this article, it was brought to the attention of *PLOS ONE* editors that there were circumstances related to this work that fall within the journal's policy on competing interests (http://www.plosone.org/static/competing.action). The Academic Editor for the manuscript, Dr. Roland Diel, has collaborated with the authors of the article on a number of recent publications. Dr. Diel was also, without his knowledge, listed as an author in an earlier version of the manuscript. Dr Diel has in the past five years received travel support and a speaker's fee from QIAGEN, the manufacturer of the QFT test. Dr. Diel holds a position at University Hospital Schleswig Holstein; the university receives support from the Institute for Statutory Accident Insurance and Prevention in the Health and Welfare Services and QIAGEN; this funding is administered by the university independently of Dr. Diel and does, according to the university, not constitute a conflict of interest in his scientific work.

Dr. Diel was invited by *PLOS ONE* to act as Academic Editor for this manuscript and we apologize that the circumstances above were not identified before he was approached to handle this manuscript.

In the light of the above, the *PLOS ONE* editors have asked an independent member of the Editorial Board, Dr. Marc Tebruegge, to evaluate the work and the peer review process of the article. This independent evaluation found that the decision to publish was appropriate in accordance with *PLOS ONE* criteria, though some clarifications and corrections as detailed below are required. The authors would therefore like to provide the following clarifications and corrections:

1. The following information was not included in the competing interests declaration for the article due to an oversight at the time of submission: The authors have read the journal's policy and declare the following conflicts: FCR has received lecture fees from Cellestis/QIAGEN. This does not alter our adherence to all the PLOS ONE policies on sharing data and materials. The other authors have declared that no competing interests exist.
2. Results Section 4.1 states, "The majority of the participants (77.4%) were female and the mean age was 38.9 years (SD 12.5). 92% of the 589 foreign born participants were born in countries with high incidence of TB." High incidence is defined as at least 40/100000.
3. Results Section 4.2 states "The average time span between the two QFT tests was 13.1 months (minimum 7 days, maximum 48.6 month) and 12.8 months (minimum 7 days, maximum 33.5 months) for the follow-up period of the 75 HCWs with a positive QFT at baseline." The average given is the median.
4. Figs 2 and 3 are missing the dashed line for the lower limit of the borderline zone; corrected figures are provided here.
5. The authors apologize for an error in syntax for the logistic regression that resulted in incorrect confidence intervals shown in Table 1. A corrected Table 1 is provided here; all confidence intervals have been corrected, and there are adjustments to some odds ratios (ORs).

**Fig 2:**
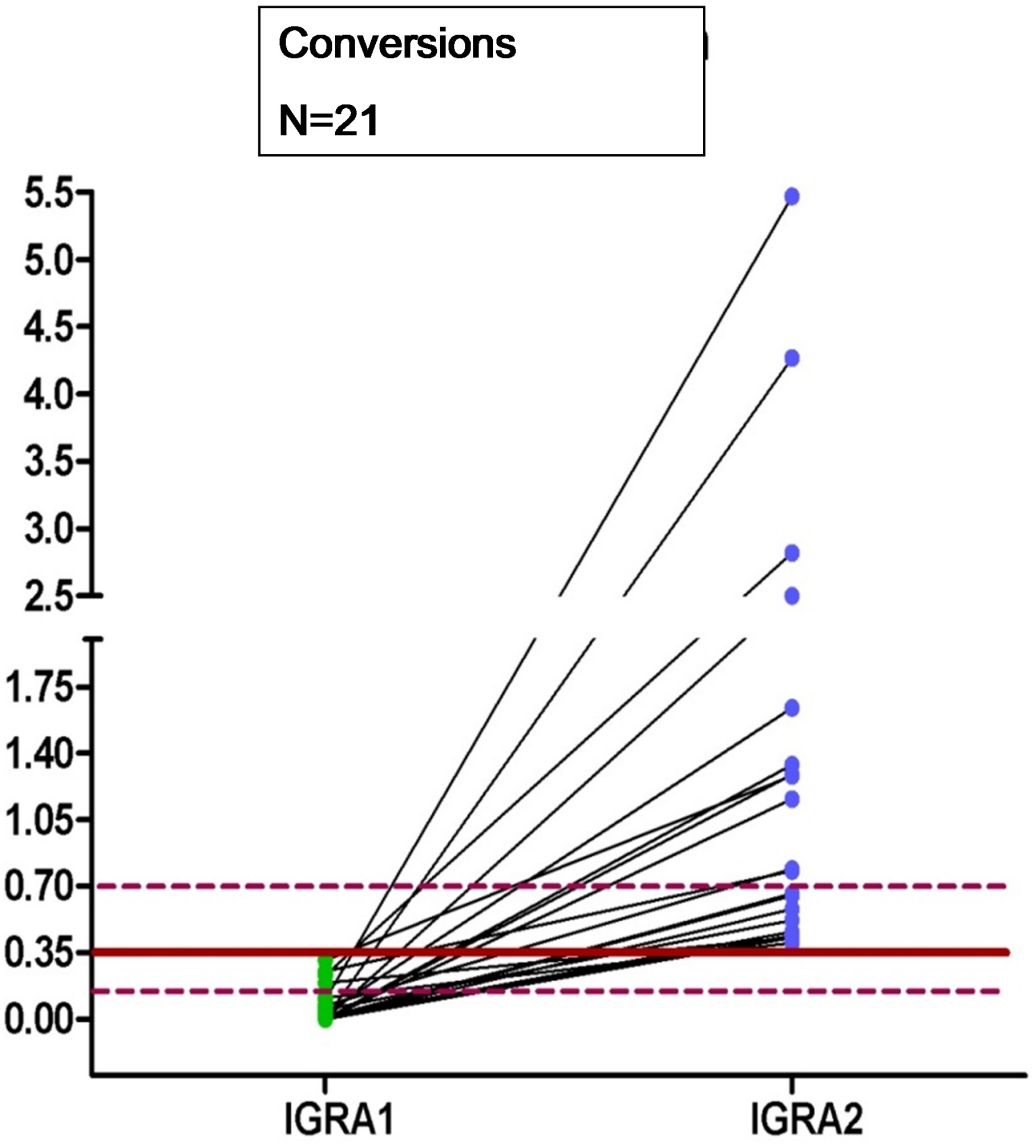
Dot plots of individual responses to QFT for conversion after the second test. The continuous line represents the cut-off 0.35 IU/ml and the dashed lines represent the borderline zone from 0.2 to <0.7 IU/ml for IFN-γ.

**Fig 3:**
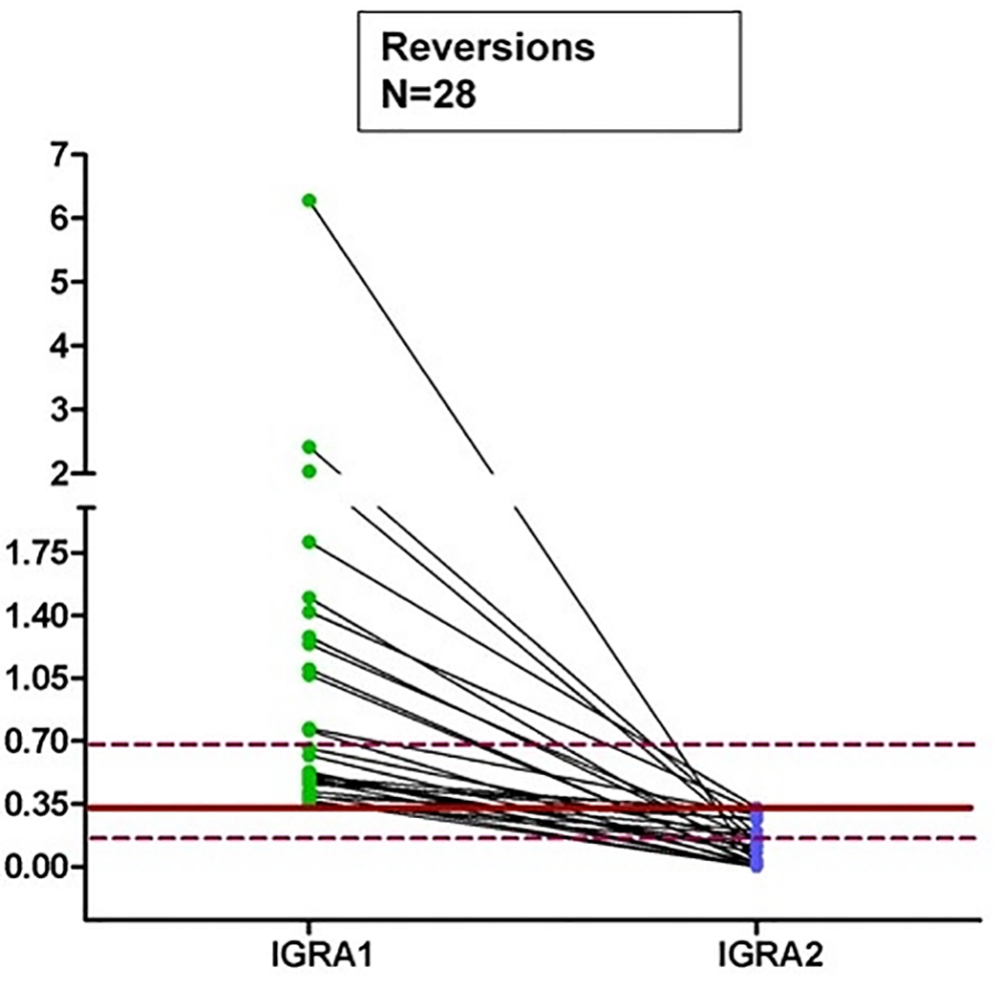
Dot plots of individual responses to QFT for reversion after the second test. The continuous line represents the cut-off 0.35 IU/ml and the dashed lines represent the borderline zone from 0.2 to <0.7 IU/ml for IFN-γ.

**Table 1:** Description of the study population and frequencies and adjusted odds ratios (OR) including 95% confidence intervals (95% CI) for covariates associated with positive QFT results.

Covariates	N/% *	QFT-IT -	QFT-T +	OR	95% CI
		n (%)	n (%)		
Age **					
<25 years	510 (13.3)	496 (97.3)	14 (2.7)	1	-
25–35 years	926 (24.2)	878 (94.8)	48 (5.2)	1.72	0.93–3.18
35–45 years	1039 (27.2)	965 (92.9)	74 (7.1)	**2.11**	**1.16–3.82**
45–55 years	975 (25.5)	868 (89.0)	107 (11.0)	**3.52**	**1.97–6.31**
>55 years	373 (9.8)	298 (79.9)	75 (20.1)	**6.89**	**3.77–12.61**
Gender**					
Female	2959 (77.4)	2716 (91.8)	243 (8.2)	1	
Male	864 (22.6)	789 (91.3)	75 (8.7)	1.29	0.97–1.73
Country of birth**					
Germany	3234 (84.6)	3012 (93.1)	222 (6.9)	1	
Foreign-born	589 (15.4)	493 (83.7)	96 (16.3)	**2.39**	**1.82–3.14**
TB in own history**					
No	3788 (99.1)	3485 (92.0)	303 (8.0)	1	
Yes	35 (0.9)	20 (57.1)	15 (42.9)	**6.25**	**3.01–12.99**
TST in history**					
no TST	1348 (35.5)	1254 (93.0)	94 (7.0)	1	
negative	1635 (42.8)	1544 (94.4)	91 (5.6)	0.74	0.54–1.01
positive	840 (22.0)	707 (84.2)	133 (15.8)	**1.99**	**1.48–2.69**
Workplace**					
Other clinical wards	610 (16.0)	577 (94.6)	33 (5.4)	1	
Internal medicine	1286 (33.6)	1190 (92.5)	96 (7.5)	1.40	0.92–2.15
Admission ward	244 (6.4)	231 (94.7)	13 (5.3)	0.90	0.45–1.80
Infection ward	389 (10.2)	355 (91.3)	34 (8.7)	**1.76**	**1.04–2.97**
Geriatric care	449 (11.7)	404 (90.0)	45 (10.0)	**1.99**	**1.21–3.25**
Radiology/Laboratory/Pathology	293 (7.7)	252 (86.0)	41(14.0)	**2.35**	**1.41–3.89**
Administration	117 (3.1)	101 (86.3)	16 (13.7)	**2.89**	**1.50–5.59**
ICU	435 (11.4)	395 (90.8)	40 (9.2)	1.50	0.91–2.49
**Profession**			Not included in the final model		
Other	302 (7.9)	277 (91.7)	25 (8.3)	1	
Physicians	583 (15.2)	538 (92.3)	45 (7.7)	0.82	0.47–1.45
Nurses	1962 (51.3)	1804 (91.9)	158 (8.1)	0,95	0.58–1.55
Administration staff	267 (7.0)	229 (85.8)	38 (14.2)	1.16	0.65–2.08
Technicians and special ward staff	222 (5.8)	200 (90.1)	22 (9.9)	0.56	0.27–1.18
Trainees	177 (4.6)	174 (98.3)	3 (1.7)	0.39	0.11–1.46
Therapist/Auxiliaries	310 (8.1)	283 (91.3)	27 (8.7)	0.91	0.49–1.68
**Reason for testing**			Not included in the final model		
Serial examination	2533 (66.3)	2310 (91.2)	223 (8.8)	1	
Contact tracing	1290 (33.7)	1195 (92.6)	95 (7.1)	0.86	0.66–1.14
**BCG vaccination**			Not included in the final model		
No	2084 (54.4)	1909 (91.6)	175 (8.4)	**1**	
Yes	1739 (45.5)	1596 (91.8)	143 (8.2)	0.89	0.69–1.15

* The final multivariate logistics model includes the variables age, gender, country of birth, TB in own history, workplace, TST

Rad/Lab/Path = Radiology, Laboratory, Pathology

As a result of this correction in the data analysis, a number of the ORs no longer reach statistical significance (specifically, Age 25–35 years, TST in history—negative, and Workplaces Internal Medicine, Admission Ward, and ICU). The Internal Medicine workplace category discussed in the article is no longer considered significant as a putative risk factor; the corrections do not affect any of the other conclusions. We therefore provide revised text with corrected ORs and removal of the Internal Medicine workplace for the relevant parts of the Abstract, Results, and Discussion sections as follows:

Abstract, paragraph 3 corrected text: We observed a prevalence of LTBI of 8.3%. Putative risk factors for a positive QFT result were age >55 years (OR 6.89), foreign country of birth (OR 2.39), personal history of TB (OR 6.25) and workplace, e.g. infection ward (OR 1.76) or geriatric care (OR 1.99). Of those repeatedly tested, 88.2% (721/817) tested consistently QFT-negative and 47 were consistently QFT-positive (5.8%). A conversion was observed in 2.8% (n = 21 of 742 with a negative first QFT) and a reversion occurred in 37.3% (n = 28 of 75 with a positive first QFT). Defining a conversion as an increase of the specific interferon concentration from <0.2 to >0.7 IU/ml, the conversion rate decreased to 1.2% (n = 8). Analogous to this, the reversion rate decreased to 18.8% (n = 9).

Results, paragraph 2 corrected text: Risk factors for a positive QFT result were an age of >55 years (OR 6.89, 95% CI 3.77–12.61), being foreign born (OR 2.39, 95% CI 1.82–3.14), TB in the individual's own history (OR 6.25, 95% CI 3.01–12.99) and workplace (Table 1). No statistically significant association was observed for the criterion of profession and reason for testing. No case of active TB was detected during the baseline screening.

Discussion, paragraph 1 corrected sentence: In our analysis, the prevalence of a positive QFT was associated with working in any kind of department with a likelihood of contact with TB patients, e.g. infection wards (OR 1.76) or Radiology/Laboratory/Pathology (OR 2.35) but also in wards with unknown TB contacts like geriatric care (OR 1.99).
